# Real-world pharmacological treatment of pregnant patients with rheumatic diseases from China: a retrospective analysis from 2016 to 2021

**DOI:** 10.3389/fphar.2024.1353293

**Published:** 2024-04-17

**Authors:** Ji-Ning Jia, Xian-Li Wang

**Affiliations:** Department of Pharmacy, The Obstetrics and Gynecology Hospital, Fudan University, Shanghai, China

**Keywords:** rheumatic diseases, pregnancy, defined daily cost, defined daily dose system, immunosuppressive agents

## Abstract

**Introduction:** We investigated trends in the use of therapeutic drugs for pregnant patients with rheumatic diseases in nine Chinese cities (Beijing, Chengdu, Guangzhou, Harbin, Hangzhou, Shanghai, Shenyang, Tianjin, and Zhengzhou) to provide a reference for drug use in clinic.

**Methods:** Outpatient prescription data for pregnant patients diagnosed with rheumatic diseases in nine cities across China in 2016–2021 were extracted from the Hospital Prescription Cooperation Project of the Hospital Pharmacy Professional Committee of the Chinese Pharmaceutical Association. A retrospective analysis was then performed, incorporating data on patient age, defined daily doses (DDDs), defined daily cost (DDC), and other metrics.

**Results:** In 2016–2020, more than 70% of the pregnant patients diagnosed with rheumatic diseases in these nine cities were 25 to < 35 years of age. The most common rheumatic diseases during pregnancy were antiphospholipid antibody syndrome (APS) and systemic lupus erythematosus (SLE). In terms of the routine use of daily therapeutic drugs, the DDDs of low molecular weight heparins (LMWHs), glucocorticoids, and immunosuppressive agents dominated the top three. Intravenous immunoglobulin (IVIG) and tumor necrosis factor inhibitors (TNFi) have been used since 2019 and had been in the forefront of the DDC.

**Conclusion:** The number and total cost of prescriptions for therapeutic drugs of pregnancy complicated by rheumatic diseases, have increased significantly over the study interval. Conventional therapeutic drugs, especially glucocorticoids, LMWHs, and hydroxychloroquine were the most widely used drugs in pregnant patients with rheumatic diseases. However, IVIG and TNFi, relatively high cost, have shown gradual increases in clinical use since 2019.

## Introduction

Rheumatic diseases are more common in females in their reproductive years. Pregnancy is a significant factor that can influence and be influenced by rheumatic diseases, including systemic lupus erythematosus (SLE), antiphospholipid antibody syndrome (APS), rheumatoid arthritis (RA), and Sjogren’s syndrome (SS) (El Miedany and Palmer, 2020). Pregnancy can potentially jeopardize both the health of the mother and the unborn child by worsening whatever rheumatic ailment the woman currently has as well as by causing fetal adverse events. Examples include recurrent spontaneous abortion in the first trimester, fetal death in the last trimester, fetal growth restriction, thrombocytopenia, preeclampsia, and eclampsia. Both the European League Against Rheumatism and the American College of Rheumatology concur that ensuring appropriate utilization of pregnancy-compatible antirheumatic drugs is imperative for attaining a satisfactory pregnancy outcome in women with rheumatic diseases (Götestam Skorpen et al., 2016; Sammaritano et al., 2020). Therefore, it is critical to continue taking rheumatic diseases medications and to understand the variety of medications that can be used to treat rheumatic diseases while pregnant (Pacini et al., 2020). At present, recommendations for safe therapeutic medications for rheumatic diseases during pregnancy have been given by guidelines from both China and abroad (Birru Talabi and Clowse, 2020; Zhang et al., 2021). Some drugs such as glucocorticoids, immunosuppressive agents, low molecular weight heparins (LMWHs), non-steroidal anti-inflammatory drugs (NSAIDs), tumor necrosis factor inhibitors (TNFi), and intravenous immunoglobulin (IVIG) can be appropriate for use during pregnancy complicated by rheumatic diseases.

The medication’s effectiveness is impacted by many factors during pregnancy, including early-pregnancy nausea and vomiting, late-pregnancy frequent urination, and changes in the body’s hormone levels. Furthermore, pregnant women are also psychologically more likely to experience negative feelings including stress, worry, sensitivity, and despair. Patients frequently have worries about the safety of medications and have poor compliance due to the specific physiological and psychological characteristics of women during pregnancy. Clinical pharmacists in hospitals should increase patient medication adherence by logical drug use promotion and enhance drug efficacy and safety monitoring. At the same time, they need to keep an eye on adverse drug reactions and drug-drug interactions, provide timely pharmacological advice to physicians and patients, and improve the level of rational drug use (Zhang et al., 2019). For example, the risk of miscarriage increases with the use of NSAIDs in the first trimester, and they should be avoided in the last stages of pregnancy because they may result in the premature closure of the fetal ductus arteriosus (Koren et al., 2006; Brouwer et al., 2015). Relatively low doses of glucocorticoids (e.g., prednisone less than 10 mg or equivalent) administered orally, intra-articularly, or intramuscularly have a better safety profile during pregnancy. However, taking more than 20 mg of prednisone per day may result in premature birth and fetal orofacial clefts (Birru Talabi and Clowse, 2020).

In this study, we statistically analyzed the use of therapeutic drugs for rheumatic diseases during pregnancy in nine Chinese cities from 2016 to 2021. We aimed to comprehensively describe the real-world prescription patterns among patients with rheumatic diseases during pregnancy by using national multi-center data sources, with a focus on comprehending patient characteristics and the trends in therapeutic drug use, in order to provide a reference for the development of rational use of clinical drugs, monitoring techniques and scientific diffusion.

## Materials and methods

### Data extraction

The research data were obtained from the Hospital Prescription Cooperation Project overseen by the Chinese Pharmaceutical Association, which incorporates 132 sample hospitals in nine cities: Beijing, Chengdu, Guangzhou, Harbin, Hangzhou, Shanghai, Shenyang, Tianjin, and Zhengzhou. Prescription data for 10 working days in each quarter from 2016 to 2021 (40 working days from 4 quarters and 240 working days in total over 6 years) were randomly selected through the information system of each hospital. Electronic information on outpatient prescriptions and inpatient medical orders over 10 working days in each quarter were summarized and included in the database of the Hospital Prescription Cooperation Project.

### Evaluation criteria and methods

Prescription data containing the fields “pregnancy” and “systemic lupus erythematosus” or “antiphospholipid antibody syndrome” or “Sjogren’s syndrome” or “rheumatoid arthritis” in the outpatient diagnosis were extracted. We extracted information on the clinical diagnosis, age, prescription number, department name, drug generic name, trade name, usage and dosage, prescription cost, drug quantity, drug unit price and other information. The research scope excluded prescriptions that only contain medications for the treatment of endocrine diseases, digestive system diseases, and other non-rheumatic diseases. The prescription data on drugs for rheumatic diseases were subjected to statistical analysis. For further analyses, we subdivided the data into four age groups (20 to < 25, 25 to < 30, 30 to < 35, 35 to < 40, ≥ 40 years).

The analysis method based on the defined daily dose system (DDDs) as recommended by the World Health Organization (WHO) (ATC/DDD Index, 2023) was adopted. Using the DDD from the 2015 Clinical Medicine Guidelines of the Pharmacopoeia of the People’s Republic of China, WHO website data and drug instructions, prescription frequency, prescribed dosage, and prescription cost were calculated for each generic name drug, and the DDDs and defined daily cost (DDC) were calculated, sorted, and analyzed. DDDs = the total annual prescribed dose of a drug/DDD of the drug. The larger the DDDs, the higher the frequency of drug usage in the clinic. DDC = the total annual prescription cost of a drug/DDDs of the drug. DDC therefore indicates the average daily cost of a drug, and a larger DDC indicates that the prescribed drugs impose a greater economic burden on patients.

### Ethics statement

This study protocol was approved by the Ethics Committee of the Obstetrics and Gynecology Hospital of Fudan University (approval number: 2023-01). The prescription data in our study solely encompassed patients age, relevant diagnosis, and utilization of treatment drugs, without any inclusion of personal identification information or medical insurance privacy. Thus, we waived the requirement for informed consent from the participants.

### Statistical analysis

We counted the types of medications used for pregnant patients with rheumatic diseases. Data classification and analyses were performed using Excel 2013 and Stata 15.0 software. We analyzed the demographic characteristics, prescription costs, and DDDs and DDC of all drug kinds in the provided data, while choosing representative drugs that were ranked in the top 10 for both DDDs and DDC for discussion respectively. Chi-square tests were used to compare demographic characters of patients and disease distribution, *p* values less than 0.05 considered as statistically significant (Pang and Ma, 2022).

## Results

### Age distribution of patients with rheumatic diseases during pregnancy

The treatment of pregnancy complicated by rheumatic diseases has attracted increasing clinical attention. The prescription number increased from 93 in 2016 to 1,544 in 2021, an increase of 1,560.2%. Pregnant women aged 25 to < 30 years and 30 to < 35 years were the main demographic who used drugs to treat rheumatic diseases, accounting for more than 70% of the surveyed prescriptions. Among them, the percentage of these patients aged 30 to < 35 years was 47.3%, 47.2%, 39.9%, 46.1%, 43.8%, and 42.0% in 2016–2021, respectively. In addition, the proportion of patients aged 35 to < 40 years gradually increased and reached a maximum in 2021, with an increase of 107.4% compared with 2016 (Table 1).

**TABLE 1:** Demographic characters and disease distribution of the pregnant women with rheumatic diseases (N = 3624).

Subgroups	2016 (N = 93)	2017 (N = 144)	2018 (N = 326)	2019 (N = 631)	2020 (N = 886)	2021 (N = 1,544)	Total (N = 3624)	*p*-vlaue^*^
Age group, N (%)								
20 to < 25	7 (7.5%)	12 (8.3%)	15 (4.6%)	19 (3.0%)	14 (1.6%)	50 (3.2%)	117 (3.2%)	< 0.05
25 to < 30	30 (32.3%)	40 (27.8%)	108 (33.1%)	190 (30.1%)	254 (28.7%)	427 (27.7%)	1,049 (28.9%)	
30 to < 35	44 (47.3%)	68 (47.2%)	130 (39.9%)	291 (46.1%)	388 (43.8%)	649 (42.0%)	1,570 (43.3%)	
35 to < 40	10 (10.8%)	19 (13.2%)	58 (17.8%)	100 (15.8%)	192 (21.7%)	346 (22.4%)	725 (20.0%)	
≥ 40	2 (2.2%)	5 (3.5%)	15 (4.6%)	31 (4.9%)	38 (4.3%)	72 (4.7%)	163 (4.5%)	
Region, N (%)								
Beijing	30 (32.3%)	21 (14.6%)	23 (7.1%)	21 (3.3%)	29 (3.3%)	59 (3.8%)	183 (5.0%)	< 0.05
Chengdu	22 (23.7%)	29 (20.1%)	48 (14.7%)	115 (18.2%)	146 (16.5%)	281 (18.2%)	641 (17.7%)	
Guangzhou	31 (33.3%)	59 (41.0%)	164 (50.3%)	355 (56.3%)	505 (57.0%)	820 (53.1%)	1934 (53.4%)	
Hangzhou	8 (8.6%)	24 (16.7%)	42 (12.9%)	70 (11.1%)	133 (15.0%)	203 (13.1%)	480 (13.2%)	
Others	2 (2.2%)	11 (7.6%)	49 (15.0%)	70 (11.1%)	73 (8.2%)	181 (11.7%)	386 (10.7%)	
disease, N (%)								
SLE	54 (58.1%)	62 (43.1%)	126 (38.7%)	188 (29.8%)	135 (15.2%)	166 (10.8%)	731 (20.2%)	< 0.05
APS	17 (18.3%)	30 (20.8%)	73 (22.4%)	249 (39.8%)	494 (55.8%)	865 (56.0%)	1728 (47.7%)	
SS	8 (8.6%)	17 (11.8%)	27 (8.3%)	33 (5.2%)	45 (5.1%)	77 (5.0%)	207 (5.7%)	
RA	7 (7.5%)	13 (9.0%)	14 (4.3%)	19 (3.0%)	33 (3.7%)	27 (1.7%)	113 (3.1%)	
Others	7 (7.5%)	22 (15.3%)	86 (26.4%)	142 (22.5%)	179 (20.2%)	409 (26.5%)	845 (23.3%)	

Abbreviation: SLE, systemic lupus erythematosus; APS, antiphospholipid antibody syndrome; SS, Sjogren’s syndrome; RA, rheumatoid arthritis.*The *p*-value of chi-square tests across subgroups.

### Prescription numbers for pregnancy with rheumatic diseases in different cities

Statistical analysis showed that the number of prescriptions varied widely among the nine studied cities from 2016 to 2021. When cities were ranked by prescription numbers, Guangzhou, Chengdu, and Hangzhou were the top three cities in the 6 years. In Guangzhou, the number of prescriptions for combined rheumatic diseases of pregnancy has been rising annually and made up more than half of the total from 2018. In addition, the number of prescriptions in Beijing has been dropping year after year, decreasing almost ten-fold from 32.3% in 2016 to 3.8% in 2021 (Table 1).

### Distribution of specific rheumatic diseases during pregnancy

We further classified and analyzed the prescription data from 2016 to 2021 to investigate the categories and characteristics of the disease spectrum in patients with rheumatic diseases during pregnancy. The percentage of prescriptions for pregnancy complicated by SLE was 58.1% in 2016, accounting for more than half of all prescriptions. This percentage gradually declined to 10.8% in 2021. The proportion of prescriptions for pregnancy complicated by APS rose annually, increasing more than three-fold from 18.3% in 2016 to 56.0% in 2021. From 2016 to 2021, the sum of prescriptions for SLE and APS accounted for more than 60% of the total each year and exceeded 70% in 2016, 2019, and 2020. By contrast, other types of rheumatic diseases, such as RA and SS, accounted for a smaller percentage of the prescriptions (Table 1).

### Total DDDs and rankings of classes of therapeutic drugs

To further explore the frequency with which various therapeutic drugs were used for pregnancy complicated by rheumatic diseases, we calculated and analyzed the DDDs of glucocorticoids, immunosuppressive agents, NSAIDs, LMWHs, plant drugs, TNFi (includes recombinant human tumor necrosis factor receptor type II-Fc fusion protein antibody and adalimumab, not other drugs such as etanercept and belimumab), and IVIG. The DDDs of therapeutic drugs increased yearly from 2016 to 2021. LMWHs, as essential drugs for the treatment of rheumatic diseases, maintained high DDDs and were consistently ranked first over the 6-year study period, followed by glucocorticoids, immunosuppressive agents, and NSAIDs. TNFi and IVIG have been used in therapy for the treatment of rheumatic diseases during pregnancy since 2019. However, their DDDs were markedly lower than those of the four previously mentioned drugs (Table 2).

**TABLE 2:** DDDs and ranking of therapeutic drugs for pregnancy with rheumatic diseases.

Classification of drugs	2016		2017		2018		2019		2020		2021	
	DDDs	Rank	DDDs	Rank	DDDs	Rank	DDDs	Rank	DDDs	Rank	DDDs	Rank
Glucocorticoids	1,545.8	2	1,946.0	2	4,523.7	2	6,512.5	2	6,812.4	3	10,078.1	4
Immunosuppressive agents	955.3	3	1,737.3	3	3,121.1	3	5,842.5	3	7,276.9	2	11,586.8	3
LMWHs	1,900.8	1	2,578.0	1	5,215.2	1	13,920.2	1	18,629.3	1	29,960.6	1
NSAIDs	205.0	4	485.0	4	1,725.0	4	4,262.0	4	6,267.0	4	11,809.3	2
TNFi	—^*^	—	—	—	—	—	27.0	6	14.8	6	14.8	6
Plant drugs	—	—	50.0	5	—	—	81.0	5	36.0	5	194.5	5
IVIG	—	—	—	—	—	—	2.0	7	1.7	7	2.3	7

* Abbreviation: DDDs, defined daily dose system; LMWHs, low molecular weight heparins; NSAIDs, non-steroidal anti-inflammatory drugs; TNFi, tumor; Necrosis factor inhibitors; IVIG, intravenous immunoglobulin.“—” means no relevant data.

### DDDs and rankings of specific therapeutic drugs

We next analyzed the top 10 specific therapeutic drugs ranked by DDDs in nine cities from 2016 to 2021. Among the LMWHs, the DDDs of dalteparin sodium were highest in 2016 and 2017, then dropped to fifth place in 2021. Conversely, enoxaparin sodium’s DDDs increased progressively from fifth in 2016 to first in 2021. The DDDs of nadroparin calcium were ranked seventh for the first 5 years of the research, and then move up one spot in 2021. Among the immunosuppressive agents, hydroxychloroquine consistently had the top three highest DDDs over the 6 years, whereas ciclosporin and azathioprine had lower DDDs. As one of the most commonly used glucocorticoids, prednisone always had high DDDs ranking and had the highest ranking in 2018. Aspirin is recognized as the most commonly used NSAIDs, and its DDDs climbed four places to second from 2016 to 2021, its highest position over the 6 years (Table 3).

**TABLE 3:** The top 10 therapeutic drugs for pregnancy with rheumatic diseases ranked by DDDs.

Rank	2016		2017		2018		2019		2020		2021	
	Generic name	DDDs	Generic name	DDDs	Generic name	DDDs	Generic name	DDDs	Generic name	DDDs	Generic name	DDDs
1	Dalteparin sodium	1,484.0	Dalteparin sodium	1,588.0	Prednisone	2,810.0	Enoxaparin sodium	9,683.0	Enoxaparin sodium	13,026.0	Enoxaparin sodium	20,408.0
2	Hydroxychloroquine	845.0	Hydroxychloroquine	1,518.6	Hydroxychloroquine	2,791.5	Hydroxychloroquine	4,914.9	Hydroxychloroquine	6,689.9	Aspirin	11,808.5
3	Prednisone	843.0	Prednisone	1,375.5	Enoxaparin sodium	2,402.0	Prednisone	4,292.0	Aspirin	6,257.0	Hydroxychloroquine	10,568.8
4	Methylprednisolone	652.8	Enoxaparin sodium	662.0	Dalteparin sodium	2,168.0	Aspirin	4,262.0	Prednisone	3,992.0	Prednisone	7,020.0
5	Enoxaparin sodium	334.0	Methylprednisolone	520.5	Aspirin	1,686.0	Dalteparin sodium	3,268.0	Dalteparin sodium	3,204.0	Dalteparin sodium	4,021.0
6	Aspirin	190.0	Aspirin	457.0	Methylprednisolone	1,683.7	Methylprednisolone	2,200.5	Methylprednisolone	2,782.9	Nadroparin calcium	3,208.8
7	Nadroparin calcium	82.8	Nadroparin calcium	328.0	Nadroparin calcium	645.2	Nadroparin calcium	913.5	Nadroparin calcium	1,692.5	Methylprednisolone	2,978.1
8	Mycophenolate mofetil	53.3	Methotrexate	120.0	Ciclosporin	159.9	Ciclosporin	441.0	Ciclosporin	377.6	Low molecular weight heparin calcium	1,283.8
9	Prednisolone	50.0	Prednisolone	50.0	Azathioprine	88.7	Azathioprine	294.7	Low molecular weight heparin calcium	372.6	Ciclosporin	692.1
10	Sulfasalazine	30.0	Leflunomide	45.0	Methotrexate	48.0	Tacrolimus	145.2	Bemiparin sodium	229.2	Fondaparinux	552.0

Abbreviation: DDDs, defined daily dose system.

### Overall prescription costs for therapeutic drugs

Next, we focused on the prescription cost data and found that the total prescription costs of therapeutic drugs for rheumatic diseases during pregnancy rose annually in nine cities from 2016 to 2021. Compared with 2016, the total cost of prescriptions in 2021 increased fourteen-fold, from 58,961.9 *yuan* to 890,133.6 *yuan*. This result demonstrates that pregnancy complicated by rheumatic diseases has been drawing increasing attention. In terms of the average prescription cost, the highest value was 634.0 ± 813.4 *yuan* in 2016, and the lowest was 464.8 ± 529.9 *yuan* in 2018 (Table 4).

**TABLE 4:** Cost of prescriptions and the average prescription cost of pregnancy with rheumatic diseases.

Year	2016	2017	2018	2019	2020	2021
Cost of prescriptions (*yuan*)	58,961.9	80,915.3	151,529.9	389,980.2	533,881.4	890,133.6
Average prescription cost (*yuan*, mean ± SD)	634.0 ± 813.4	561.9 ± 687.6	464.8 ± 529.9	618.0 ± 645.7	602.6 ± 641.5	532.9 ± 554.6

### DDC and rankings of classes of therapeutic drugs

We compared the costs of various drugs used from 2016 to 2021 by DDC analysis. LMWHs and immunosuppressive agents had the first and second highest DDC from 2016 to 2018. By contrast, IVIG and TNFi surpassed immunosuppressive agents and LMWHs from 2019 to 2021, with the top two DDC rankings, as these drugs were introduced for the treatment of pregnancy complicated by rheumatic diseases in 2019 and were relatively expensive. It is especially noteworthy that the DDC of IVIG was much higher than that of other drugs (Table 5).

**TABLE 5:** DDC and ranking of therapeutic drugs for pregnancy with rheumatic diseases.

Classification of drugs	2016		2017		2018		2019		2020		2021	
	DDC (*yuan*)	Rank	DDC (*yuan*)	Rank	DDC (*yuan*)	Rank	DDC (*yuan*)	Rank	DDC (*yuan*)	Rank	DDC (*yuan*)	Rank
Glucocorticoids	0.9	3	0.6	5	0.8	3	0.7	6	0.8	6	0.6	6
Immunosuppressive agents	16.5	2	11.6	2	12.6	2	14.7	4	14.0	4	12.7	4
LMWHs	21.9	1	22.9	1	20.3	1	19.4	3	22.3	3	24.1	3
NSAIDs	0.6	4	0.9	4	0.6	4	0.5	7	0.5	7	0.5	7
TNFi	—^*^	—	—	—	—	—	727.6	2	126.9	2	99.4	2
Plant drugs	—	—	4.4	3	—	—	4.1	5	4.3	5	6.4	5
IVIG	—	—	—	—	—	—	3,322.0	1	3,360.0	1	3,501.4	1

Abbreviation: DDC, defined daily cost; LMWHs, low molecular weight heparins; NSAIDs, non-steroidal anti-inflammatory drugs; TNFi, tumor necrosis factor inhibitors; IVIG, intravenous immunoglobulin.*“—” means no relevant data.

### DDC and rankings of specific therapeutic drugs

Tacrolimus, as representative immunosuppressive agents, had the highest DDC rankings from 2016 to 2018 but fell back in 2019–2021. The DDC of hydroxychloroquine gradually decreased from sixth in 2016 to 10th in 2019 and was no longer in the top 10 in 2020 and 2021. Similarly, nadroparin calcium’s DDC ranking declines annually, falling from third in 2016 to 10th in 2021, while the ranking for enoxaparin sodium falls from fourth to outside the top 10. The use of IVIG and TNFi was the main cause for the lower DDC rankings of the above drugs in 2019–2021. Human immunoglobulin and recombinant human tumor necrosis factor receptor type II-Fc fusion protein antibody had the highest DDC values in these 3 years. The DDC of glucocorticoid was not among the top 10 from 2016 to 2021 (Table 6).

**TABLE 6:** The top 10 therapeutic drugs for pregnancy with rheumatic diseases ranked by DDC.

Rank	2016		2017		2018		2019		2020		2021	
	Generic name	DDC (*yuan*)	Generic name	DDC (*yuan*)	Generic name	DDC (*yuan*)	Generic name	DDC (*yuan*)	Generic name	DDC (*yuan*)	Generic name	DDC (*yuan*)
1	Tacrolimus	115.6	Tacrolimus	116.7	Tacrolimus	90.9	Human immunoglobulin	3,322.0	Human immunoglobulin	3,360.0	Human immunoglobulin	3,501.4
2	Mycophenolate mofetil	56.1	Ciclosporin	47.4	Ciclosporin	45.8	Recombinant human tumor necrosis factor receptor type II-Fc fusion protein antibody	727.6	Recombinant human tumor necrosis factor receptor type II-Fc fusion protein antibody	727.6	Recombinant human tumor necrosis factor receptor type II-Fc fusion protein antibody	320.0
3	Nadroparin calcium	34.3	Nadroparin calcium	31.9	Mycophenolate mofetil	33.6	Tacrolimus	80.9	Fondaparinux	126.5	Fondaparinux	134.0
4	Enoxaparin sodium	29.5	Enoxaparin sodium	27.1	Nadroparin calcium	33.5	Mycophenolate mofetil	49.4	Bemiparin sodium	122.6	Bemiparin sodium	117.3
5	Dalteparin sodium	19.6	Dalteparin sodium	19.3	Enoxaparin sodium	20.4	Ciclosporin	48.9	Tacrolimus	83.8	Tacrolimus	91.3
6	Hydroxychloroquine	13.1	Hydroxychloroquine	11.7	Dalteparin sodium	17.1	Nadroparin calcium	30.8	Adalimumab	83.4	Adalimumab	83.4
7	Leflunomide	10.3	Leflunomide	6.0	Hydroxychloroquine	10.5	Low molecular weight heparin calcium	22.2	Ciclosporin	47.1	Ciclosporin	40.4
8	Ibuprofen	3.0	Celecoxib	5.4	Diclofenac sodium	8.6	Enoxaparin sodium	19.3	Nadroparin calcium	28.2	Mycophenolate mofetil	33.7
9	Loxoprofen	2.9	Compound glycyrrhizin	4.7	Etoricoxib	7.2	Dalteparin sodium	16.4	Low molecular weight heparin calcium	24.6	Parnaparin sodium	30.2
10	Sulfasalazine	2.2	Total glucosides of paeony	4.3	Azathioprine	4.0	Hydroxychloroquine	10.4	Enoxaparin sodium	20.3	Nadroparin calcium	25.4

Abbreviation: DDC, defined daily cost.

## Discussion

The incidence of rheumatic diseases is relatively high during pregnancy, and pregnancy and rheumatic diseases affect each other. Rheumatic diseases can easily lead to infertility, miscarriage, premature birth, and other adverse pregnancy outcomes, and pregnancy also aggravates rheumatic diseases. Therefore, long-term drug therapy to maintain disease stability is particularly important for improving the quality of life for pregnant women (Østensen et al., 2015; Giles et al., 2019). Based on statistical data, we analyzed real-world trends in medication for pregnancy complicated by rheumatic diseases in nine cities from China over the 6 years.

The prevalence of rheumatic diseases is higher between the ages of 15 and 50, and to investigate the relationship between age and disease in demographic characteristics, we divided the age of patients in the prescription data into five groups: 20 to < 25, 25 to < 30, 30 to <35, 35 to < 40, and ≥ 40 years (Borchers et al., 2010). Notably, the proportion of patients aged 35 to <40 years gradually increased to more than double during the study period from 2016 to 2021. This phenomenon was likely to be related to the comprehensive “two child policy” issued by China in October 2015 and to the fact that the number of older pregnant women has also been increasing annually (Li et al., 2019). In addition, SLE and APS are the two prevalent rheumatic diseases observed in pregnant women, which is consistent with the epidemiological studies (Mekinian et al., 2015; Pons-Estel et al., 2017). Our analysis results showed that the number of prescriptions for pregnancy complicated by rheumatic diseases increased yearly. This may imply that the prevalence or visiting rates of pregnant patients with rheumatic diseases increased over time. In terms of drug therapy, traditional antirheumatic drugs such as glucocorticoids, LMWHs, and immunosuppressive agents were the most popular drugs.

LMWHs can reduce the risk of pregnancy complications, including unexplained recurrent pregnancy loss, fetal death, and intrauterine growth restriction (Satta and Biondi, 2019; Schreiber and Hunt, 2019). Dalteparin sodium, enoxaparin sodium, and nadroparin calcium are regarded as basic therapeutic drugs that meet the health requirement of patients. In particular, there are clear beneficial effects of LMWHs combined with low-dose aspirin for rheumatic diseases during pregnancy (Fernandes et al., 2020). Low-dose aspirin, which is a classic representative drug of NSAIDs, can not only be used in patients with APS but also protects SLE patients from hypertensive diseases during pregnancy (Hoffman et al., 2020). Interestingly, a retrospective study revealed that NSAIDs use in the first trimester may raise the incidence of abortion and malformations (Li et al., 2018), with the exception of low-dose aspirin. Aspirin is being used more frequently in clinic, and our analysis of the data indicated that its DDDs climbed to second place in 2021.

Glucocorticoids are classic antirheumatic drugs that have been used clinically for a long time. A number of management standards and practices for pregnancy complicated by rheumatic diseases from China and abroad recommend glucocorticoids as a class of major antirheumatic drugs (Flint et al., 2016a; Flint et al., 2016b; Götestam Skorpen et al., 2016; Zhang et al., 2021). Pregnancy may be considered in women with rheumatic diseases who use no more than 10 mg of prednisone daily to maintain the stability of the disease. If disease activity increases during pregnancy, we can consider appropriately increasing the dosage of glucocorticoids or combining them with relatively safe immunosuppressive agents (Zhang et al., 2021). Recently, hydroxychloroquine has been explored as a widely used immunosuppressive agents that stabilizes the endothelium and has antithrombotic effects (Misra et al., 2020). Its continuous use is generally advised during pregnancy to enhance disease protection and improve pregnancy outcomes (Sammaritano et al., 2020). Sulfasalazine and azathioprine are also widely used for rheumatic diseases during pregnancy (Viktil et al., 2012; Flint et al., 2016a). These drugs are considered relatively safe when used as directed. For example, sulfasalazine may be used at a dosage of less than 2 g/d together with folic acid (5 mg/d) supplementation throughout the whole period of pregnancy (2019). In addition, the lowest effective dose of tacrolimus or ciclosporin can be used during pregnancy and has not been associated with fetal malformation. However, it is necessary to perform increased monitoring of the patient’s own indicators, such as blood pressure, kidney function, and blood drug concentration (Zhang et al., 2021).

TNFi and IVIG were gradually more widely used in the clinic in 2019–2021. Owing to the high economic cost of these drugs, as evidenced by their DDC being significantly higher than that of glucocorticoids, LMWHs, immunosuppressive agents, and NSAIDs, their DDDs were much lower than those of traditional antirheumatic drugs and had lower acceptance from patients. A clinical study showed that there was no increase in adverse events during pregnancy after IVIG administration (Flint et al., 2016a), suggesting that it can be safely used to treat rheumatic diseases during pregnancy. However, the correlation between TNFi and adverse events during pregnancy has been inconsistent among different reports. On one hand, previous studies have shown that TNFi can be used during early pregnancy without increasing the incidence of adverse pregnancy events and neonatal infections (Diav-Citrin et al., 2014). Etanercept and pexelizumabare, characterized by low placental transport rates, are also considered to be safe throughout pregnancy (Götestam Skorpen et al., 2016). Current evidence suggests that exacerbations in RA patients during pregnancy is significantly associated with refraining from TNFi treatment upon positive pregnancy tests. Therefore, considering the risk of disease onset and adverse pregnancy outcomes, it is recommended to continue TNFi treatment during pregnancy (Gerardi et al., 2022). On the other hand, it has also been reported that the use of TNFi during pregnancy is likely to carry some risks, and it has been recommended that the use of TNFi be stopped for at least 12 weeks before pregnancy (Carter et al., 2009). Notably, a growing number of plant drugs have gradually become the choice for the treatment of pregnancy complicated by rheumatic diseases; these include total glucosides of paeony and the compound glycyrrhizin. Some individual patients who were diagnosed with pregnancy complicated by rheumatic diseases did not choose ongoing pregnancy owing to special circumstances, and they were eventually exposed to drugs classified as category X by the United States Food and Drug Administration, such as methotrexate and mycophenolate mofetil. To summarize, the types of prescription drugs we extracted from the prescription data conform to the selection scope of drugs for the treatment of pregnancy complicated by rheumatic diseases recommended by guidelines from China and abroad.

As the number of prescriptions for pregnancy complicated by rheumatic diseases increased annually from 2016 to 2021, the total sample prescription costs also increased. However, the average prescription cost decreased from 2016 to 2018, reaching the lowest level in 2018, and this may be related to the update of the medical insurance catalogue and the implementation of canceling the drug price addition in China (National Health Commission of the People’s Republic of China, 2017).

There were a few limitations in our study. Firstly, the research prescription data were randomly chosen within 10 business days of each quarter, and given that the prescriptions we analyzed were from municipalities or provincial capitals in China, some economically depressed regions may have varied prescription patterns due to varying medical and economic issues. As a result, our study might not accurately represent the state of medication use among pregnant Chinese women with rheumatic diseases as a whole. Secondly, our analysis was conducted based on prescription data which only mentioning “pregnancy” and “systemic lupus erythematosus” or “antiphospholipid antibody syndrome” or “Sjogren’s syndrome” or “rheumatoid arthritis” in the diagnostic information. Consequently, some prescriptions of outpatients with rheumatic diseases of pregnancy might be excluded due to their diagnostic information not matching the above search terms. Thirdly, limited information on prescription data and lack of patient medical history limited the evaluation of rationality. Finally, our study was based on outpatient sampling prescriptions, and pregnancy outcomes and neonatal follow-up of pregnant women with rheumatic diseases were lacking. In the future, further collecting prescription samples with more complete information and wider geographical coverage is necessary, and further investigations are indispensable to analyze the correlation between the efficacy of drugs and their possible adverse side effects. In addition, the specific usage and dosage information of each patient should be accurate, as well as the safety and rationality of medication should be evaluated in combination with the pregnancy outcome of pregnant patients and relevant guidelines.

## Conclusion

Our results revealed firstly the prescription patterns of pregnant women with rheumatic diseases over the last 6 years in China. The number and total cost of prescriptions for pregnancy complicated by rheumatic diseases in nine cities increased yearly from 2016 to 2021. Traditional drugs such as glucocorticoids, LMWHs, and hydroxychloroquine are the most frequently choices for pregnant patients with rheumatic diseases in China due to their apparent efficacy and cost-effectiveness. Significantly, the high cost of IVIG and TNFi may be one factor in the lower use of them compared with conventional therapeutic drugs, but they also play a role in treating rheumatic diseases of pregnancy that cannot be substituted by other drugs. In order to maximize therapeutic effect of IVIG and TNFi, we should always pay attention to the patient’s condition, although the current experimental data suggest that these drugs are deemed safe for pregnant women.

## Data Availability

The original contributions presented in the study are included in the article, further inquiries can be directed to the corresponding author.
